# Economics of Philanthropy

**Published:** 1898-03-05

**Authors:** 


### The Institutional Workshop.

### ECONOMICS OF PHILANTHROPY.

### III.—OLD AND NEW SYSTEMS.

It might be supposed that the famous Act of the Speenhamland justices, whatever might be thought of it from the point of view of economics, at least made the poor comfortable. So, doubtless, the Berkshire magnates expected; but the result proved them wrong. This "giving that as charity which was due for work," as Professor Thorold Rogers calls it, never went beyond the donation of a bare subsistence; and while the workman who had his quiver full obtained this, the earnings of the single or the childless one, who got only a small allowance or none, were hardly enough to support life. Here are some statistics of that time, taken from the same writer's "Work and Wages": In Hunts the average wages were 9s. 3d. per week; in Herts, 12s. 6½d.; in Norfolk, 11s. 3d. A Yorkshire labourer earned less than 6s. a week, his wife 1s., and his child something infinitesimal. And this was at a time when wheat was at 104s. a quarter. What parish allowance could raise this up to a "living wage"?

Sentiment often makes laws; but it is oftener selfishness that administers them; and while the first moves only by fits and starts, the second is a permanent force. Landlords who owned a whole parish pulled down every cottage upon their estate. They got their labour at the cheap current rate, while the parish in which the labourer lived and had his settlement, as the phrase ran, had to add the necessary supplement to his wages, and keep him after he was unfit for work. This law of settlement was a great misfortune for the English labourer. Depending as he did upon parish aid, he was afraid to leave the parish where by birth, apprenticeship, or long residence, he had gained a settlement and the right to relief, for fear he might not gain one in another. For this reason men would refuse to leave a parish where they were earning next to nothing, even when they were offered remunerative work in another district. Nor can we blame them when we read how, if a stranger fell ill, he was taken off at once to his own parish, even though he was in so weak a state that his life was endangered by the journey. No wonder that the labourer lost courage and energy. He was a serf, bound to the soil, and afraid to go outside his own bounds lest he should lose the dole that kept him from the starvation from which his own work was powerless to save him. No wonder that a writer* has said: "All the injury inflicted upon the labouring classes by the deliberately hostile legislation of Plantagenet or Tudor statesmen was but as dust in the balance compared with what they suffered from the benevolent measures of some of the best men that have ever ruled in England."

We have thought it useful to detail at some length this century-old experiment in humanitarianism, because it shows so forcibly "how not to do it," how not to improve the condition of the poor. What the able-bodied poor want is not alms but work, and the remuneration that work has a right to. So much does material welfare depend on the moral quality behind, that it has been found that slave labour, or that labour which is performed only as an excuse for the receipt of charity, is infinitely inferior to free labour. The labourers on relief works are not as energetic as these same men would be if they were employed on an ordinary job, from which they would be dismissed if they proved incompetent. Work alone is not enough; it must have in it the elements of hope, of proportionate reward, of permanent improvement in the condition of the worker. The men whom the Speenhamland justices tried to help profited nothing by their good intentions, because it ended in their being paupers, and having no hope of being anything else. Hopeless poverty is always lazy. Various writers on economics make the same remark, that a century ago the Scotch were famous for their idleness as to-day they are famous for their determined industry. Their country was a poor one, and they themselves had little hope of escaping from lifelong poverty. Each writer gives his own explanation of the undeniable fact that Scotland is now a prosperous country, and that its inhabitants are industrious, and it may be of use to state these explanations, and inquire how much truth there is in each. Hearn, in his "Plutology," gives the credit to the long lease system; Mr. McLeod, in his book on "Banking," to the cash credits given by the Scotch banks; while he also gives due weight to the widespread system of national education, in which Scotland was a century ahead of the rest of the kingdom.

The importance of the last is very obvious. With education a man can compete for posts which would never be open to him otherwise. In Scotland the commercial and professional ranks have long been recruited from the peasant and artisan classes to an extent which is unknown elsewhere; and this being so in one generation has inevitably helped it to be so in the next. The Church in Scotland has always been a democratic body, belonging to the people and sympathising with them, and anxious to help the more clever among them to rise to a higher social and financial position. "That state of life to which it has pleased God to call him" never appears to a Scotchman to be inevitably that in which he was born. But the other circumstances must be taken into consideration. The cash-credit system enables a young man who has no capital, and little chance of getting any by inheritance or otherwise, to set up in business for himself, if only he can find someone with enough faith in his industry and capacity to guarantee his bank account for a moderate sum. As it is the interest of the guarantor to see that his *protégé* does not too long delay the repayment of the sum guaranteed, and equally the interest of the young man to show his patron that he is reliable, so that if at any time he wants security for a larger amount it may be granted him, there has rarely been any loss through this practice, which is by no means a common one in England, and is, we believe, absolutely forbidden by the regulations of the Bank of France. The responsibility of the guarantee is often divided among several of the friends of the youth who wishes the cash-credit; so that the joint security of one or two only moderately wealthy

* T. W. Fowle, M.A. "The Poor Law," in the English Citizen Series, chap. iii., p. 67.

individuals often gives a man his first start up the ladder of life.

The advantages of the long lease in agriculture are obvious. A tenant will not expend money on improving land unless he has a fair assurance that he himself will reap the benefit of his efforts. An uncertain tenure always makes poor farming. It is a matter for regret that a blow has been struck at the long lease system by a measure conceived in the popular interest. Since the repeal of the game laws, landlords have been much less willing to grant long leases than formerly. Their only security against having their game destroyed lies in their power to evict at the end of the year a tenant who has done so. So little can the effect of a well-intentioned law be predicted. Farmers are rarely poachers; and it is a pity that the tenure of an industrious man should be endangered by the foolish prank of a boy or servant. Doubtless, in the end some arrangement between landlord and tenant will be arrived at; we only note the immediate effect, as showing that bad results may follow from the best intentions. In both the causes which are given as tending to the improvement of the people of Scotland, the real motive-power has been the chance of rising to a better position than was enjoyed previously, and this lies at the root of the matter. Any philanthropic experiment, however well-intentioned, which seeks to make the recipients contented with their lot in life, may indeed be successful, but only by making them lazy and dependent instead of active and self-reliant.

